# Dynamic survival prediction of end-stage kidney disease using random survival forests for competing risk analysis

**DOI:** 10.3389/fmed.2024.1428073

**Published:** 2024-12-11

**Authors:** Daniel Christiadi, Kevin Chai, Aaron Chuah, Bronwyn Loong, Thomas D. Andrews, Aron Chakera, Giles Desmond Walters, Simon Hee-Tang Jiang

**Affiliations:** ^1^Department of Immunology and Infectious Disease, John Curtin School of Medical Research, Australian National University, Canberra, ACT, Australia; ^2^Department of Renal Medicine, The Canberra Hospital, Garran, ACT, Australia; ^3^Centre of Personalised Medicine, Australian National University and Canberra Health Services, Canberra, ACT, Australia; ^4^School of Population Health, Curtin University, Perth, WA, Australia; ^5^Research School of Finance, Actuarial Studies & Statistics, Australian National University, Canberra, ACT, Australia; ^6^Department of Renal Medicine, Sir Charles Gairdner Osborn Park Health Care Group, Nedlands, WA, Australia; ^7^Australian National University Medical School, Garran, ACT, Australia

**Keywords:** dynamic prediction model, end-stage kidney disease, landmarking, random survival forests, competing risk

## Abstract

**Background and hypothesis:**

A static predictive model relying solely on baseline clinicopathological data cannot capture the heterogeneity in predictor trajectories observed in the progression of chronic kidney disease (CKD). To address this, we developed and validated a dynamic survival prediction model using longitudinal clinicopathological data to predict end-stage kidney disease (ESKD), with death as a competing risk.

**Methods:**

We trained a sequence of random survival forests using a landmarking approach and optimized the model with a pre-specified prediction horizon of 5 years. The predicted cumulative incidence function (CIF) values were used to generate a personalized dynamic prediction plot.

**Results:**

The model was developed using baseline demographics and 13 longitudinal clinicopathological variables from 4,950 patients. Variable importance analysis for ESKD and death informed the creation of a sequence of reduced models that utilized six key variables: age, serum albumin, bicarbonate, chloride, eGFR, and hemoglobin. The models demonstrated robust predictive performance, with a median concordance index of 84.84% for ESKD and 84.1% for death. The median integrated Brier scores were 0.03 for ESKD and 0.038 for death across all landmark times. External validation with 8,729 patients confirmed these results.

**Conclusion:**

We successfully developed and validated a dynamic survival prediction model using common longitudinal clinicopathological data. This model predicts ESKD with death as a competing risk and aims to assist clinicians in dialysis planning for patients with CKD.

## Introduction

Chronic kidney disease (CKD) is a global health challenge, with a reported prevalence of 13.4% (1), and is associated with significant mortality (2) and morbidity (3). As patients approach end-stage kidney disease (ESKD), clinicians and patients face complex decisions regarding the optimal timing for permanent dialysis access formation and evaluating transplantation suitability. These decisions are further complicated by the highly variable and often unpredictable rate of CKD progression. Therefore, an accurate model to predict ESKD is vital.

The Kidney Failure Risk Equation (KFRE) (4) is widely recognized as the gold-standard predictive model for ESKD. It is based on a Cox-proportional hazard model that uses a combination of baseline clinicopathological predictors. Similar to KFRE, other studies have explored various machine learning models and neural networks that rely on baseline patient characteristics and clinicopathological data as predictors (5–9).

However, these static predictive models have notable limitations. They often fail to account for the variability and non-linear relationships between predictors and ESKD over an extended follow-up period, which is critical given the chronic nature of the disease. Additionally, they may miss valuable prognostic information derived from changes in these variables over time. For example, the estimated glomerular filtration rate (eGFR) has been demonstrated to exhibit highly variable trajectories among patients with CKD (10). Despite this variability, time-varying eGFR remains a critical predictor of ESKD and all-cause mortality (11, 12). Consequently, static predictive models are primarily applicable to new patients at baseline rather than to those being monitored during follow-up (13).

To incorporate time-varying covariates, Chuah et al. and Wang et al. featurised time-series data into features to predict ESKD as a binary classification problem (14, 15). While this approach demonstrated superior predictive performance, using binary classification to address a time-to-event problem introduces certain limitations.

First, this method potentially sacrifices the interpretability and flexibility of modeling event probabilities as a function of time. Additionally, it does not account for patients lost to follow-up or censored, nor does it consider the impact of competing events such as death. In populations with CKD, which are often characterized by older age and chronic comorbidities, failing to account for death can result in inaccurate ESKD risk predictions (16).

Several publications have introduced dynamic prediction models to address the limitations of static approaches (13, 17–20). In statistical literature, a clinical predictive model capable of processing longitudinal data to update predictions over time is referred to as a dynamic prediction model. Not only would this methodology mimic a nephrologist’s approach to patient evaluation, but the methodology has also been shown to improve model fit and performance (19).

There are two primary methods for dynamic prediction: joint models (21, 22) and landmarking (23–25). Joint models generate predictions by linking a Cox proportional hazard model with linear mixed models to account for time-varying covariate trajectories. However, correctly specifying a joint statistical model for all covariate trajectories may be challenging or unstable, and incorporating additional time-varying covariates may be computationally expensive (26–28).

In contrast, the landmarking approach is more straightforward and better equipped to handle multiple time-varying covariates. Picket et al. proposed combining random survival forests with the landmarking approach, effectively bypassing the limitation of Cox models, including the proportional hazards assumption (29). Simulation studies demonstrated the superior performance of this approach over joint models and Cox-based landmarking in capturing complex relationships between survival outcomes and longitudinal covariates.

Our study builds on this technique, extending it to analyze multiple longitudinal clinicopathological data points to predict ESKD while accounting for death as a competing event. The model has been externally validated and offers the potential for clinical decision support in real-world settings.

## Methods

### Study population

This observational, time-to-event study included all adult patients aged over 17 years (*N* = 10,198) who attended a single tertiary nephrology unit at the Canberra Hospital, a university hospital in Australia. Clinicopathological data, encompassing both inpatient and outpatient data, along with patient demographics, were extracted from an internal database covering the period from 1st September 1996 to 14th January 2022 (Supplementary Table 1). Patients with pre-existing ESKD (defined as those undergoing chronic dialysis or with a history of kidney transplantation) were excluded from the analysis.

For external validation, a cohort of patients aged ≥18 years (*N* = 8,729) was obtained from a Western Australian pathology provider, spanning data from 1st January 2006 to 21st April 2022. This external cohort was prepared using the same exclusion criteria and filtering methodology as the internal cohort (see Results). The study was conducted in accordance with the Declaration of Helsinki and was approved by the ACT Health Research Ethics and Governance Office (ETHLR.18.040). Patient consent was waived per the National Statement on Ethical Conduct in Human Research (2007, Chapter 2.3).

### Events and censoring definition

The primary event of the study was ESKD, with death from all causes considered a competing event. ESKD was defined as a sustained eGFR value less than or equal to 15 mL/min/1.73m^2^ or the commencement of chronic dialysis or kidney transplantation. Specifically, this required evidence of persistent eGFR <15 mL/min/1.73m^2^ without subsequent recovery (eGFR >17 mL/min/1.73m^2^) during the follow-up period or the commencement of dialysis, including peritoneal dialysis, hemodialysis, hemodiafiltration, or nocturnal hemodialysis.

The first recorded eGFR value marked the start of the observation period (designated as time 0). Age at presentation was defined as the patient’s age at time 0. Observed time was measured from time 0 to the earliest event (ESKD or death) or to censoring. For patients who did not experience an event, the follow-up duration was censored at the data extraction date.

### Statistical analysis

The normality of continuous data was assessed using the Shapiro–Wilk test. For normally distributed data, differences between groups were evaluated using analysis of variance (ANOVA) and summarized as mean ± standard deviation (SD). For non-parametric data, the Kruskal-Wallis test was used to assess group differences, and results were summarized as median ± interquartile range (IQR). Pairwise comparisons between two groups were conducted using either the *t*-test or the Mann–Whitney *U* test, depending on the normality of the data. Values of *p* < 0.05 were considered significant, and all tests were two-tailed.

### Dataset imputation

Within the internal cohort, only eGFR and demographic data were complete at baseline (time 0), while all other clinicopathological predictors had a median missing value rate of 18% (Supplementary Table 2). During model training and performance analysis, missing baseline values in the training set (time 0) were imputed using the R package “mice” (version 3.14.0) with the “defaultMethod” set to predictive mean matching. After imputing missing baseline values, missing values for each variable at subsequent time points were filled out using the most recent entry.

For the test dataset, missing values were filled exclusively using the most recent entry. No additional imputation was performed to preserve the reliability of the prediction performance and to reflect clinical practice, where clinicians often rely on prior available data when results are unavailable.

For the external dataset, data cleaning was conducted to address errors and inconsistencies in patient demographic data (e.g., birth dates and sex). Due to the high rate of missing test values (Supplementary Table 2), serum glucose was imputed using a combination of previous and future data entries. In contrast, all other clinicopathological test values were filled exclusively using the last available observation for each patient.

### Dataset splitting

We evaluated performance using a 5-fold event-stratified cross-validation approach. The dataset was divided into training and test sets for each fold in an 80:20 ratio. The training set was used to train the model, while model performance was evaluated on the corresponding test set.

### Model development

As proposed by Van Houwelingen, the landmarking method applies a survival model to patients who are still at risk at the time of interest, a pre-specified landmark time (23, 24). In this method, the survival model is fitted as a function of predictors measured up to the landmark time. By selecting a series of landmark time points, the method produces a sequence of survival predictions focused on events occurring in the medically relevant timeframe, known as the prediction horizon. A detailed explanation of this approach is provided in the Supplementary material.

We implemented the landmarking approach with pre-specified landmark times at 0.5, 1, 1.5, 2, 2.5, and 3 years, with a prediction horizon of 5 years. These landmark times were chosen because stable chronic kidney disease patients are typically reviewed in the clinic every 6 months. Following the recommendation of the landmarking method’s originator, administrative censoring was applied to individuals who had not experienced an event by the end of each landmark prediction horizon.

Instead of the Cox-proportional hazard model, we used a random survival forests. The random survival forests is a non-parametric, ensemble, tree-based method (30). The algorithm trains multiple survival trees to analyze right-censored survival data.

The model uses a weighted log-rank splitting rule based on Gray’s test (31) and calculates the cumulative incidence function (CIF) to ensure accurate risk prediction in the presence of competing risks (16). Additional advantages of the model include its ability to model non-linear effects and interactions among high-dimensional predictors and its robustness in vases where the proportional hazard assumption is violated (29, 32, 33).

We implemented the built-in function in the R package “randomForestSRC” to tune the random survival forests model. The tuning process was performed to find the optimal *“mtry,”* the number of predictors that should be included as candidates for splitting, and *“nodesize,”* which represents the number of unique cases in each terminal node, determined based on the out-of-bag prediction error rate. We used the default parameters for “*mtryStart*,” which equals the number of predictors divided by 2, and *“nodesizeTry”* of (1, 2, 3, 4, 5, 6, 7, 8, 9, 10, 15, 20, 25, 30, 35, 40, 45, 50, 55, 60, 65, 70, 75, 80, 85, 90, 95, 100). Then, we used the optimal *“mtry”* and *“nodesize”* based on the out-of-bag error. We set the number of trees at 1,000.

We used baseline data—including gender, diagnosis of glomerulonephritis or vasculitis, and initial age at presentation—and longitudinal, time-varying clinicopathological data as predictors. The diagnoses of glomerulonephritis or vasculitis were verified through kidney biopsy. All other diagnoses were inputted as missing values. We then compared the performance of the standard landmark approach, consisting of adjusting the survival model on the last observed value of the biomarkers before each landmark time (LOCF) or based on the predicted value at the beginning of each landmark time obtained from the linear mixed model (34, 35). The linear mixed model is the standard approach in analyzing repeated measures and dependent data of longitudinal biomarkers. We modeled the predicted longitudinal clinicopathological data values using baseline measurements and the timing of these measurements, applying either an unconditional growth model (LME) or an unconditional quadratic growth model (LMEpoly).

The details of the R package version and the codes used in the analysis are posted at https://github.com/daniel-christiadi/dynamic_prediction_ESKD.git.

The trained models can be downloaded from https://drive.google.com/drive/folders/1yj7McUimsEsYxZjuMG26-oELjY3oMFmf?usp=sharing.

### Model performance

Model performance analysis in the internal cohort was performed on the test dataset obtained using a 5-fold cross-validation approach and measured on each pre-specified landmark time described above. Model discrimination was reported as the concordance index (C-index), which measures the ratio of subjects with the worse predicted outcome that fail earlier than other subjects in a randomly selected pair of subjects to the number of all available subject pairs (32). A higher concordance index indicates superior model discrimination, with a concordance index of 50% representing a random prediction.

The parsimonious model (Top 5) performance was also analyzed using the area under the time-dependent ROC curve (td-AUC) (36). The predicted value of the random survival forests for each landmark was assessed at the pre-specified prediction horizon. Similar to the concordance index, a higher td-AUC indicates superior model discrimination.

Model discrimination and calibration were assessed using the integrated Brier score (iBS) (37), calculated from the start of each landmark time to the prediction horizon. Unlike the C-index, lower iBS values indicate better model performance, with an iBS below 0.25 considered indicative of a useful model.

Performance on the external validation cohort was performed using models trained on the entire internal dataset, which was then evaluated on all external datasets. Due to the lack of death data in the external cohort, we could only report the external ESKD error rate.

To calculate the performance of our model against the 8-variable KFRE, we filtered the internal dataset. Only patients who had three or more serum measurements of albumin, bicarbonate, chloride, eGFR, hemoglobin, phosphate, calcium, and urine albumin-creatinine ratio, which are the combined variables used by our models and the 8-variable KFRE. Then, the dataset was manipulated to have death-censored outcomes to accommodate KFRE. To allow a fairer comparison, we input the value of the covariates at the last landmark time into the KFRE formula (38) to predict ESKD at 2 and 5 years from the last landmark time, which were the same times of interest for our model.

All analyses were performed using R 4.3.1 (39).

### Dynamic prediction plot

As the output of the trained model, we created a dynamic prediction plot. The predicted CIF values from each model for the events of interest were aggregated by each time point (from year three to year eight) using the arithmetic mean to draw the CIF curves. Higher percentages indicate a greater likelihood of the event occurring. To create the dynamic prediction plot, readers are recommended to follow the steps described at https://github.com/daniel-christiadi/dynamic_prediction_ESKD.git.

### Sensitivity analysis

We performed two sensitivity analyses. First, we created a dataset (named “sens”) with a different censoring definition. Instead of using the definition mentioned previously, for patients with no ESKD or death event, we censored the subjects on the last recorded clinicopathological test date. Furthermore, we incorporate only the tests within the previous 10 years from events or the final clinicopathological tests (the alternative censoring definition). The date of the earliest eGFR within the 10 years became time 0, and the initial age at presentation was calculated from this time point.

Additionally, we created another dataset with an additional three or more urine albumin/creatinine ratio (uACR) filter criteria (named “acr3”). To reduce the missing value, we converted the urine protein/creatinine ratio to uACR according to the published formula (40).

## Results

### Dataset creation and baseline characteristics

Of the 10,198 patients, 15 of them were excluded due to incomplete baseline demographic information (absence of gender and birth year). Another 495 patients were removed due to pre-existing ESKD, and two patients were excluded due to incorrect dates (the initial clinicopathology test occurred after the documented death date), leaving 9,686 patients with 4,458,014 individual clinicopathological values for further analysis.

Some patients exhibited skewed test frequencies, likely due to recurrent hospitalisations, prolonged stays, or frequent clinic visits (Supplementary Figure 1A). Therefore, we aggregated (resampled) the daily test results into mean monthly values to prevent this cohort from skewing the results (Supplementary Figure 1B). Due to the uneven data density of the available clinicopathological tests and to ensure longitudinal data availability, we filtered patients on three or more of the following criteria: hemoglobin, total white cell count, platelet count, CKD-EPI-based eGFR, and serum measurement of sodium, potassium, chloride, bicarbonate, calcium, phosphate, albumin, alkaline phosphates, and glucose, leaving 4,950 patients (Supplementary Figure 1C). This dataset will now be referred to as dense3.

The median initial age of the study population was 62 (IQR 47 to 73), with a median baseline eGFR of 50 mL/min/1.73m^2^. The patients had a median follow-up time of 7 years (IQR 3 to 13). Only 500 of 4,950 patients were documented to have any diagnosis of glomerulonephritis or vasculitis. ESKD events occurred in 1,270 (25%) and death in 733 (15%) patients. In the external cohort, the total cohort size was 8,729 subjects with a median age of 57 (IQR 47 to 66). The patients had relatively similar follow-up durations. Notably, patients in the external cohort had better baseline kidney function (median eGFR 89 mL/min/1.73m^2^). Due to the absence of death information, only an ESKD event was reported. A total of 246 patients of 8,729 (3%) experienced ESKD. The baseline demographic and clinicopathological data from the internal and external cohorts are displayed in Table 1.

**Table 1:** Baseline summary of internal and external cohorts.

Variables	Dense3 (*N* = 4,950)	Acr3 (*N* = 2,916)	External cohort (*N* = 8,729)
Demographic data			
Gender - Female	2,217 (45%)	1,280 (44%)	4,865 (56%)
Initial age of presentation (median, IQR)	62 (47–73)	59 (45–70)	57 (47–66)
Glomerulonephritis or vasculitis diagnosis	500 (10%)	445 (15%)	No information
Follow-up time in years (median, IQR)	7 (3–13)	8 (4–14)	8 (5–13)
Event			
ESKD	1,270 (25%)	639 (22%)	246 (3%)
Death	733 (15%)	278 (9.5%)	No information
Censored	2,947 (60%)	1,999 (69%)	8,483 (97%)
Baseline clinicopathological data			
Serum albumin in g/L (median, IQR)	42 (39–44)	42 (39–44)	43 (41–45)
Serum alkaline phosphatase in U/L (median, IQR)	78 (63–97)	77 (63–95)	73 (60–89)
Serum bicarbonate in mmol/L (median, IQR)	23 (21–26)	24 (22–26)	28 (26–29)
Serum calcium in mmol/L (median, IQR)	2.36 (2.27–2.44)	2.36 (2.27–2.43)	2.37 (2.31–2.44)
Serum chloride in mmol/L (median, IQR)	105 (102–107)	105 (102–107)	103 (101–105)
eGFR in mL/min/1.73m^2^ (median, IQR)	50 (30–73)	57 (36–77)	89 (74–90)
Serum glucose in mmol/L (median, IQR)	5.7 (5–7.20)	5.6 (5–7.3)	5.3 (4.8–5.9)
Hemoglobin in g/L (median, IQR)	133 (118–146)	136 (122–148)	139 (129–149)
Serum phosphate in mmol/L (median, IQR)	1.13 (0.99–1.28)	1.12 (0.99–1.26)	1.10 (1–1.22)
Platelet count in x10^9^/L (median, IQR)	240 (198–290)	245 (203–294)	255 (212–305)
Serum potassium in mmol/L (median, IQR)	4.2 (3.9–4.6)	4.3 (4–4.6)	4.3 (4.1–4.6)
Serum sodium in mmol/L (median, IQR)	139 (137.5–141)	140 (138–141)	140 (139–141)
White cell count in x10^9^/L (median, IQR)	7.3 (6–8.9)	7.3 (6–8.8)	6.6 (5.4–8.2)
Albumin creatinine ratio in mg/mmol (median, IQR)	NA	13 (2–92)	NA

### Models comparison

**Figure 1:**
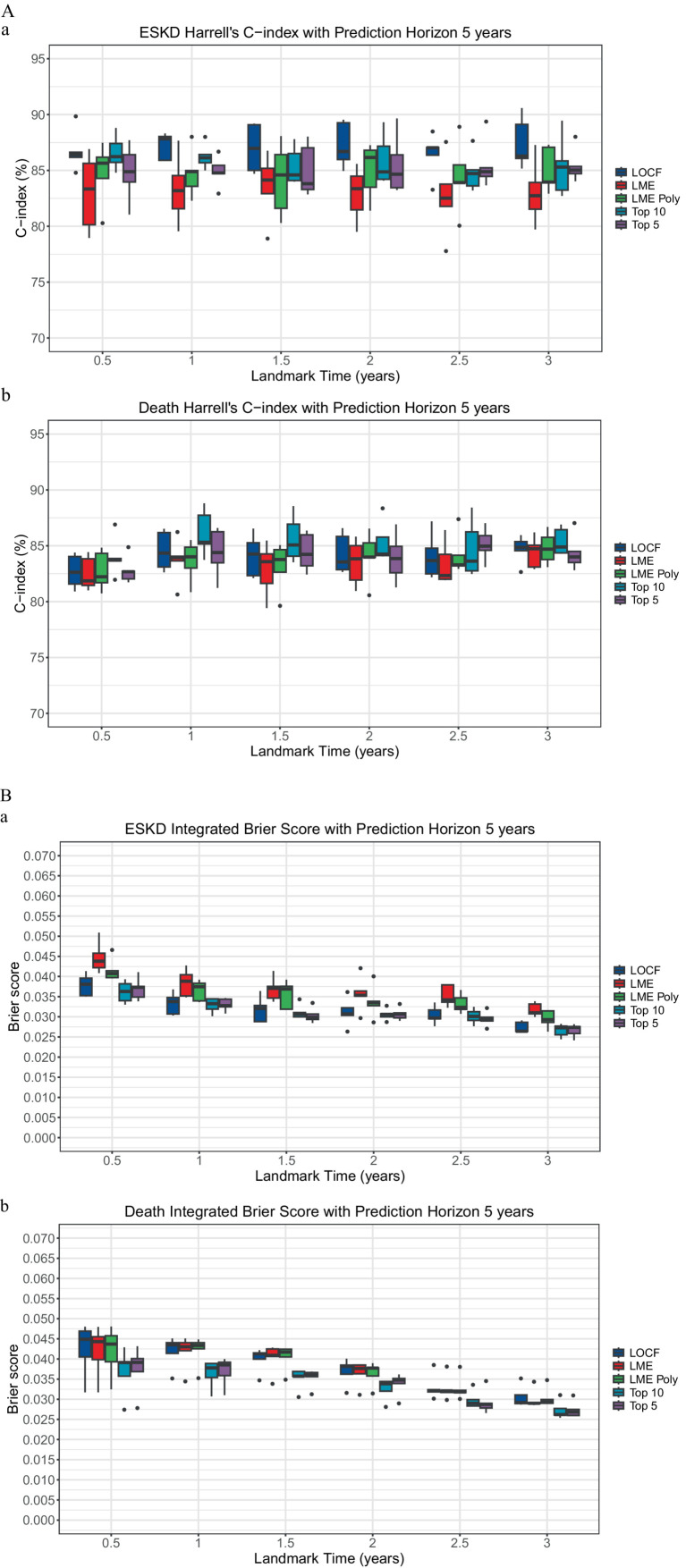
Comparison of landmarking approach. **(A)** The Concordance index. **(a)** ESKD: The Kruskal–Wallis test comparing the ESKD concordance index of the models for each landmark time was non-significant. Each boxplot was created using the model’s performance on the five-fold cross-validation test dataset. **(b)** Death: The Kruskal–Wallis test comparing the death concordance index of the models for each landmark time was non-significant. Each boxplot was created using the model’s performance on the test dataset of the five-fold cross-validation. **(B)** Integrated Brier score. **(a)** ESKD: The Kruskal-Wallis test comparing the ESKD integrated Brier score of the models for each landmark time was non-significant. Each boxplot was created using the model’s performance on the test dataset of the five-fold cross-validation. **(b)** Death: The Kruskal–Wallis test comparing the death-integrated Brier score of the models for each landmark time was non-significant. Each boxplot was created using the model’s performance on the test dataset of the five-fold cross-validation.

To incorporate longitudinal data, the landmarking approach can utilize the last observed value of the longitudinal biomarkers, known as the last observed carried forward (LOCF), or incorporate all the previous longitudinal biomarkers to model the value of the respective biomarkers at the beginning of each landmark time. The linear mixed model is the standard approach in analyzing repeated measures and dependent data of longitudinal biomarkers. Two linear mixed model methods were used for comparison: the unconditional growth model (LME) and the unconditional quadratic growth model (LME poly). At each landmark time, there was no significant performance difference between models that used time-varying clinicopathological data value using the LOCF, LME, and LME poly approach in predicting ESKD or death (Figure 1 and Supplementary Table 3).

### Important predictors

**Figure 2:**
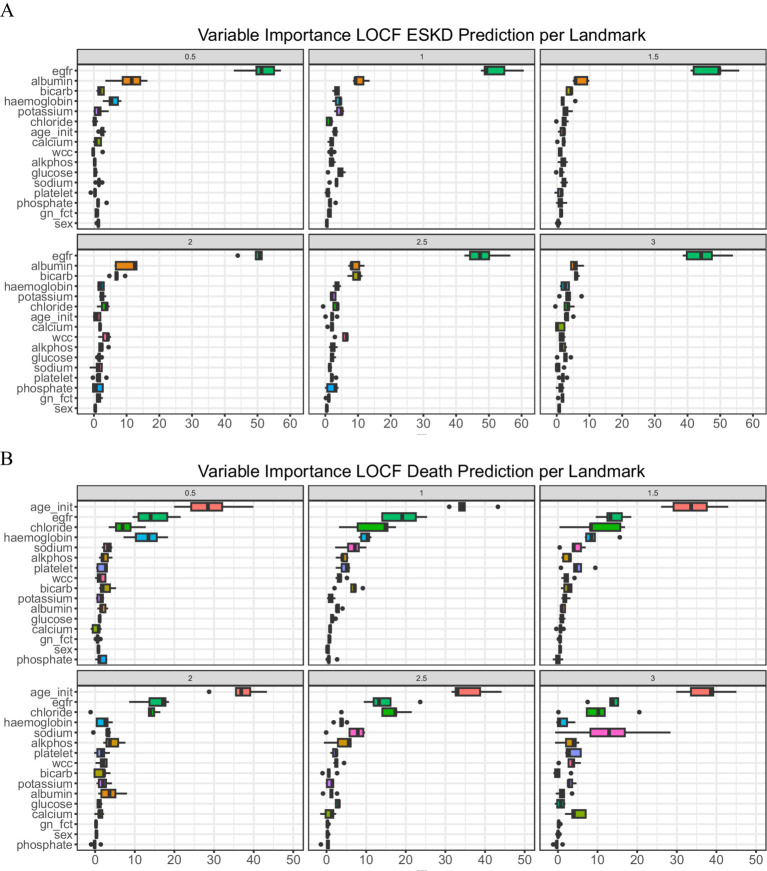
Variable of importance. **(A)** ESKD and **(B)** Death. A bar plot value indicates median with an interquartile range.

To examine the importance of each covariate in the model prediction, we explored the variable importance (VIMP, Figure 2). A large VIMP indicates the model relies on the respective predictors to achieve prediction accuracy and is thus likely a potentially predictive variable. We calculated the median of variable importance for individual predictors using 5-fold cross-validation and the interquartile range. eGFR is the most important predictor for ESKD prediction in all landmark times, followed by serum albumin and bicarbonate. In contrast, initial age at presentation, eGFR, and serum chloride are crucial for death prediction. To test whether we could reduce the number of predictors to allow visualization of the dynamic prediction plot, we combined the VIMP value for ESKD and death for all the landmark times and calculated their median (Supplementary Table 4).

We then compared the performance of the full model (LOCF) against a sequence of models using the top 10 (Top 10) longitudinal predictors (serum measurement of albumin, alkaline phosphatase, bicarbonate, chloride, potassium, and sodium, eGFR, hemoglobin, platelet count, and white cell count) and a sequence of models using the top five longitudinal predictors (serum measurement of albumin, bicarbonate, chloride, eGFR, and hemoglobin). For the top 10 and top 5 models, we removed the diagnosis of glomerulonephritis or vasculitis and gender due to low median VIMP values.

The ESKD concordance index for the top 5 models was close to 85%, except at the 1.5-year landmark, where the median was 83.81% (IQR: 83.3 to 87.02%, Supplementary Table 3A), with a median td-AUC for ESKD of approximately 84.9% (Supplementary Table 3C). Additionally, the ESKD iBS for the top 5 was approximately 0.03 (Supplementary Table 3B). No significant performance differences were found when comparing the top 5 models to the full model or the top 10 models (Figure 1), indicating the top 5 variables accounted for most of the model’s predictive capability.

### External validation performance

In external validation, the ESKD median concordance index across all landmark times ranged from 83.1 to 88.3% (Supplementary Table 3). The Wilcoxon rank-sum test comparing the ESKD concordance index at each landmark between the top 5 models and external validation showed no significant difference.

### Comparison to 8-variable KFRE

Using the clinicopathological data of death-censored patients in the test datasets on the last landmark time, the 8-variable KFRE model (38) was used to calculate ESKD prediction at 2 years and 5 years. There is no performance difference between our models and KFRE at 5 years, but the 8-variable KFRE concordance index at 2 years was superior (Supplementary Table 6).

### Dynamic prediction plot

**Figure 3:**
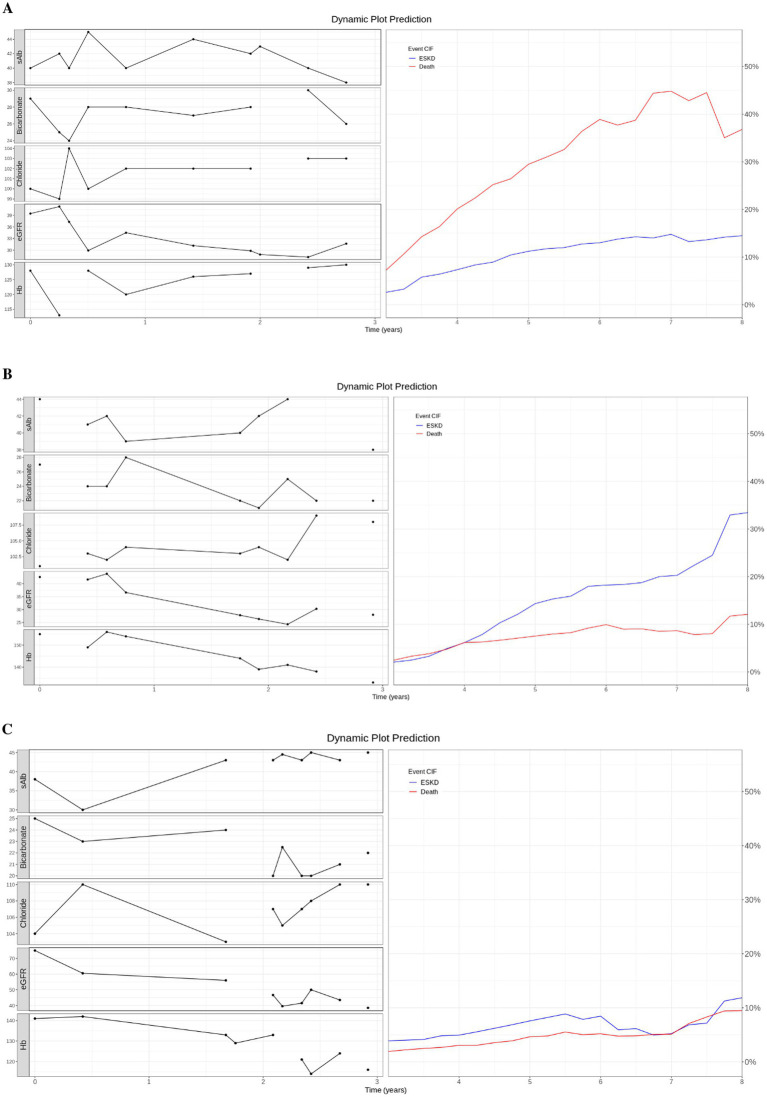
Dynamic prediction plot. **(A)** Case 1: An 80-year-old patient died at 7.2 years. **(B)** Case 2: A 62-year-old patient with ESKD event at 5.08 years. **(C)** Case 3: A 67-year-old patient did not experience any event and was censored at 6.69 years. **(A–C)** On the left of the dynamic prediction plot, we plot all the observed longitudinal clinicpathological data (serum measurement of albumin, bicarbonate, chloride, eGFR, and hemoglobin) for the first 3 years. On the right, we drew the predicted CIF curve for ESKD and Death using the top 5 trained models on patients from the test dataset.

We used three case studies to demonstrate the utility of the dynamic prediction plot (Figure 3). For case 1, an 80-year-old patient had the last clinicopathological test at 6.91 years and died at 7.2 years. The predicted death CIF curve peaked at 7.5 years. Case 2, a 62-year-old subject, was followed for 11.66 years with an ESKD event at 5.08 years. The ESKD CIF curve steadily rose and overtook the death curve from 4 years onwards. Finally, a 67-year-old patient was followed until 6.69 years without any event. ESKD and death CIF curves peaked at approximately 10%.

### Sensitivity analysis

To create a “sens” dataset, 10,198 patients were extracted. A total of 15 patients were excluded due to incomplete baseline demographic information (absence of gender and birth year). Another 495 patients were removed due to pre-existing ESKD. A total of 31 patients did not have any clinicopathological tests within 10 years of the event, resulting in 9,657 patients for further analysis. Applying the same filter criteria as for ‘dense3’, the ‘sens’ dataset included 4,225 patients. To create “acr3,” we applied additional filter criteria (three or more uACR) to the dense3 dataset, resulting in 2,916 patients.

There was no significant difference in discrimination or calibration performance between the original, conventional censoring definition (LOCF, censoring date based on the data extraction date) and the alternative censoring definition (sens, censoring date based on the last clinicopathological data). There was no improvement in performance when the urine albumin/creatinine ratio was included in the acr3 model training (Supplementary Figure 2 and Supplementary Table 5).

## Discussion

We demonstrated how longitudinal clinicopathological data can be incorporated to create a dynamic prediction plot that estimates the risks of death and ESKD. The graph, designed as an end-user interface, resembles the workflow of specialist clinical practice. CKD patients are typically monitored quarterly to annually, generating dense clinicopathological data points over extended follow-up periods. In contrast, static predictive models require nephrologists to disregard valuable data points or previous predictions, treating each visit as if the patients were new.

Instead of relying on cross-sectional values, clinicians could use all accumulated and updated data points to generate personalized dynamic predictions. These predictions can be used during patient education, decision-making regarding dialysis preparation, and resource planning, especially for older patients with underlying poor kidney function who are at risk for both events. By integrating clinical expertise with the predicted risks provided by dynamic predictions, clinicians can better guide discussions about whether patients at high risk of death should undergo dialysis preparation, such as fistula creation.

Our limited comparison showed that our model performed similarly to KFRE at predicting ESKD at 5 years and was inferior to KFRE at 2 years. However, this is not a valid comparison because the KFRE was designed using baseline data and provided a static prediction at a single time point. The KFRE model was not designed to be applied to subsequent clinicopathological data. Moreover, KFRE was created from a cohort of patients with eGFR less than 60 mL/min/1.73m^2^ with a development cohort comprised of patients with a mean age of 70 years (71% of patients were more than 65 years old) to predict ESKD without taking death into account. Of all patients aged 70 years or older in our cohort (“dense3”), 495 patients of 1,612 (31%) died before developing ESKD, and 423 (26.2%) experienced ESKD as the first event. Therefore, analyzing the performance of KFRE in our cohort will likely produce biased results. Our model seeks to move to an analysis of continuous data and outputs estimates of both ESKD and death to better inform clinical decision-making.

We also observed a higher prevalence of ESKD (25%) during the study period in the internal cohort. To compare the event, we reviewed datasets from another state in Australia (Tasmania). Irish et al. reported 2.2% ESKD and 6.8% death within 2 years of observation (41). The difference is likely due to the data source. Our cohort came from patients referred to nephrologists with CKD. TASlink was a cohort created through data linkage. It is also worth pointing out that our external validation cohort (from Western Australia) had a relatively similar ESKD prevalence of 3%, and the parallel performance we demonstrated to the external cohort implied the external validity of our models.

The difference in ESKD prevalence potentially affected the comparison of internal versus external performance. The median external ESKD concordance index at the landmark time of 0.5 years was better than the internal ESKD concordance index. However, it had a wider interquartile range and did not achieve statistical significance in formal comparison (*p* = 0.056). Reviewing the respective landmarks, 489 patients in the internal cohort had ESKD events, while only 50 ESKD events occurred in the external cohort. In addition, unlike the internal cohort used to train the algorithm, our validation cohort did not have mortality information. As a result, we could not estimate the C-index for death and the integrated Brier score.

Our experiment showed no performance difference between the standard landmarking approach that used the LOCF approach versus modeling the clinicopathological values using linear mixed models. Our findings further emphasized the difficulties of modeling real-life biomarker trajectories using linear mixed models observed in the literature (27, 28). This finding might extrapolate to the joint model’s likely suboptimal performance, as the approach combines a Cox model with linear mixed models that estimate the trajectories of longitudinal biomarkers (35).

Furthermore, random survival forests with landmarking can tackle the limitation of restrictive proportional hazard assumptions, outperform joint modeling or Cox landmarking in the setting of a complex relationship between the survival and longitudinal biomarkers (29), and are also less sensitive to misspecification of longitudinal biomarker trajectories (34).

The strength of our study lies in the incorporation of high-dimensional, longitudinal clinicopathological data with a landmarking approach and a random survival forests capable of predicting ESKD with death as a competing risk. The models were built with a relatively large dataset and externally validated in a larger dataset in another Australian center. Another advantage of our approach is that it utilizes common clinicopathological data and does not require a urine albumin/creatinine ratio.

However, this approach has several limitations. We had to train six different models as we pre-specified six landmark times, requiring significant computing resources. Extending the landmark times would increase the entire model’s size and limit the model’s applicability. To ensure the models provide a continuous prediction for patients with accumulated data points over 3 years, we could apply the models as a rolling window. For example, patient Mr. X had clinicopathological data repeated for 4 years. Our model cannot process the clinicopathological data after 3 years and can only provide a prediction of ESKD or death up to 8 years when, theoretically, we have the information to predict Mr. X’s outcome at 9 years. Therefore, we disregard the longitudinal data of the preceding 1 year and use the clinicopathological data at year 1 as input for the landmark 0.5-year model. As a result, this also means longitudinal data starting from 1.5 years will be used for the landmark 1-year model, longitudinal data from 2 years for the landmark 1.5-year model, and data from 2.5 years for the landmark 2-year model. Applying the approach will allow the model to provide an extended prediction when the patients have repeated tests after 3 years of follow-up. To support this approach, our sensitivity analysis showed similar performance of models trained using the conventional time 0 definition (first kidney function test) versus using the earliest kidney function test within 10 years of events (“sens,” Supplementary Figure 2 and Supplementary Table 5).

Another limitation is that we could only categorize the etiology as glomerulonephritis or vasculitis, while other ESKD aetiologies—such as diabetes, hypertension, post-nephrectomy, or other causes—had to be grouped into an “others” category. A kidney biopsy is required to definitively diagnose glomerulonephritis or vasculitis. However, biopsies are not always mandatory and can sometimes be relatively contraindicated, as in the case of single kidneys.

For patients with underlying conditions such as diabetes and hypertension, which are highly prevalent globally, determining the exact cause of ESKD can be particularly challenging. Furthermore, the diagnosis category was identified as having low importance for model performance (Figure 2). We suspect that the varying kidney or mortality outcomes associated with different underlying conditions could be better predicted by the model using longitudinal data, such as eGFR or other biomarker measurements, rather than relying on baseline ESKD etiology.

Finally, it is crucial that we test the model performance in patients with reduced kidney function following an intervention, such as post-nephrectomy (42) or cryoablation (43), in future studies. We expect the longitudinal model, similar to ours, will have the advantage when predicting the outcomes, as the model can incorporate multiple sequential biomarker values before and after the procedure, and the cohort might experience some improvement in kidney function due to hypertrophy of the remaining kidney tissue.

In conclusion, we developed and validated a dynamic survival prediction model to predict ESKD, incorporating death as a competing risk. The model utilized common longitudinal clinicopathological data and demonstrated performance in satisfactory discrimination and calibration.

## Data Availability

The raw data supporting the conclusions of this article will be made available by the authors, without undue reservation.
