# Supplementary material for: Dynamic survival prediction of end-stage kidney disease using random survival forests for competing risk analysis
Source: Front Med (Lausanne). 2024 Dec 11;11:1428073. doi: 10.3389/fmed.2024.1428073 (PMC11668785; doi:10.3389/fmed.2024.1428073)
Supplement: Supplementary file 1 [file Data_Sheet_1.docx]

**Supplementary Materials**

The observed data is given by $D_{n}=\{T_{i}, \delta_{i}, x_{i}, y_{i};i=1, .., n\}$ where $T_{i}=min(T_{i}^{*}, C_{i})$ is the observed time for the $i$-th subject $(i=1, \ldots, n)$ with $T_{i}^{*}$ denoting the true event time, $C_{i}$ the consoring time, and $\delta_{i}=I(T_{i}^{*}\leq C_{i})$ as the event indicator. $x_{i}$ is the baseline, static covariates, and $y_{i}$ is continuous vector of longitudinal measurements for the biomarker of interests with $y_{ij}= y_{i}(t_{ij})$ denoting the value of the biomarker measured at time $t_{ij}$ for $j=1, \ldots, n_{i}$, where $n_{i}$is the number of biomarker repeatedly measured on individual $i$.

We aim to predict the survival probability of a new individual $m$ based on his/her accumulated longitudinal biomarkers and baseline covariates at the prediction horizon $\tau+s, s>0$, given $m$ is event-free at landmark time $\tau$, and $s$ is pre-specified window of interest. Hence, dynamic prediction is defined

$$\pi_{m}\left( \tau+s \right| \tau, x_{m}, \bar{y}_{m}(\tau))=Pr(T_{m}^{*}\geq\tau+s | T_{m}^{*}> \tau, D_{n},x_{m}, \bar{y}_{m}(\tau)$$

To achieve the aim, Van Houwelingen proposes the landmarking method. The method incorporates a sequence of survival models for a subsample of subjects still at risk at a specific time, known as landmark time. Hence, the subsequent landmark time model will include only a subset of subjects that "survive" or event-free from the preceding landmark time^23,24^.

Interest is often expressed in creating many landmark times, which leads to the creation of a prediction model at each time. Each landmark time model can be fitted to predict the occurrence of event of interest in a relevant, pre-specified time frame, known as the prediction horizon, $\tau+s$.

Any biomarker information accrued up to each pre-specified landmark time is incorporated as an additional predictor, $\bar{y}_{m}(\tau)$^23,24^. The naive landmark approach utilises the last-observed values of the biomarkers as $\bar{y}_{m}(\tau)$ (name LOCF model in our manuscript). Another approach incorporates the different times of biomarker measurement and its potential measurement error by predicting the value of the biomarker using a linear mixed model (LME and LME poly model in our manuscript)^35^.

To reduce bias, the author of the approach recommends that subjects still event-free at the prediction horizon be censored^23,24,34^.

Classically, a Cox proportional hazards model is used. However, Picket et al. introduced the combination of a landmarking approach with random survival forests, a popular machine-learning (ML) algorithm^29^. Random survivalforests is a non-parametric survival model, which offers the capability of processing high-dimensional data, detecting non-linear relationships and interactions. The method implemented same general principles as random forests: (a) Survival trees are grown using bootstrapped data; (b) Random feature selection is used when splitting tree nodes; (c) Trees are generally grown deeply, and (d) The survival forest ensemble is calculated by averaging terminal node statistics.

We utilised random survivalforests implemented in R package “randomForestSRC”. The package extends the random survival forests to analyse competing risk using modification of Gray’s test^44^. The estimators of the CIF are calculated within the terminal nodes of a random survival forests tree and then aggregated to form the ensemble. Further details regarding the splitting rules, event-specific ensemble can be found in the original paper^30,32^.

At each landmark time, the competing risk random survival forests algorithm is applied to the respective landmark dataset to build a dynamic risk prediction ensemble.

**Supplementary Figures**

Figure 1. Heatmap of frequency of clinicopathological data

1A. Heatmap of original data


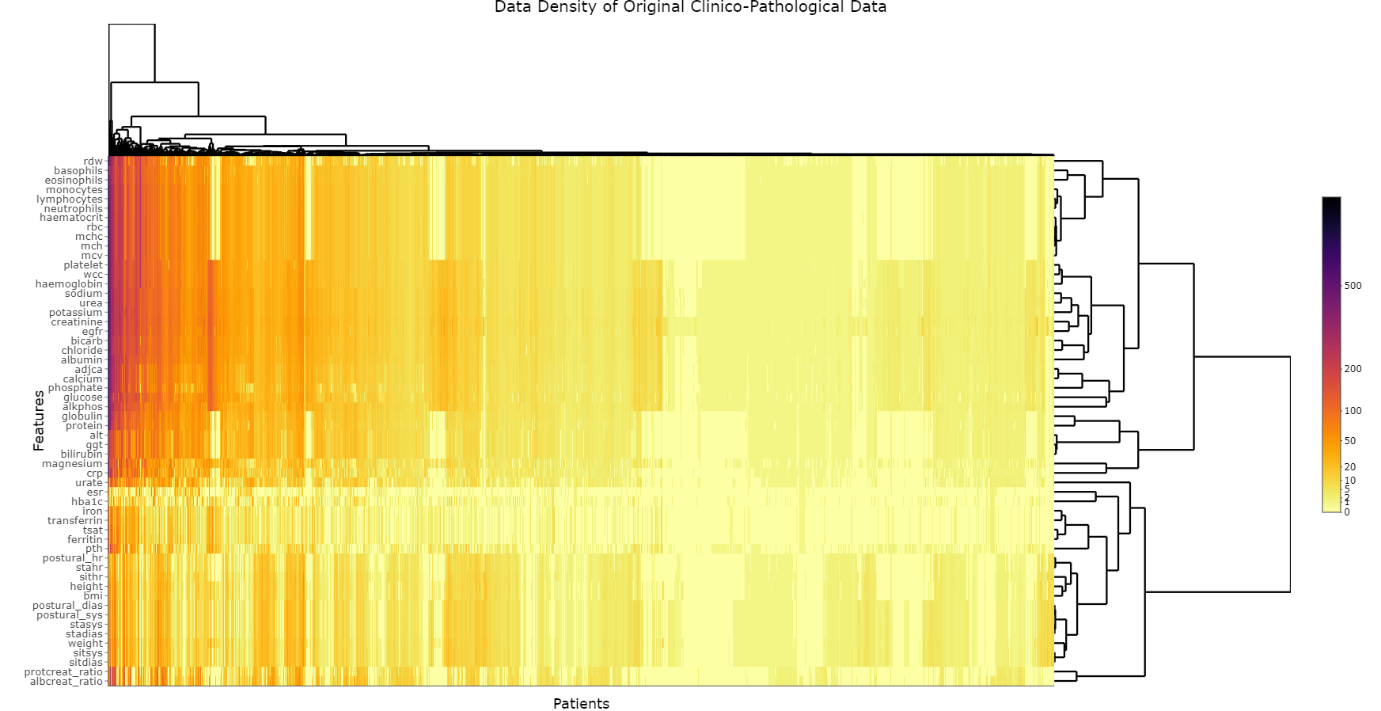


1B. Heatmap of resampled data


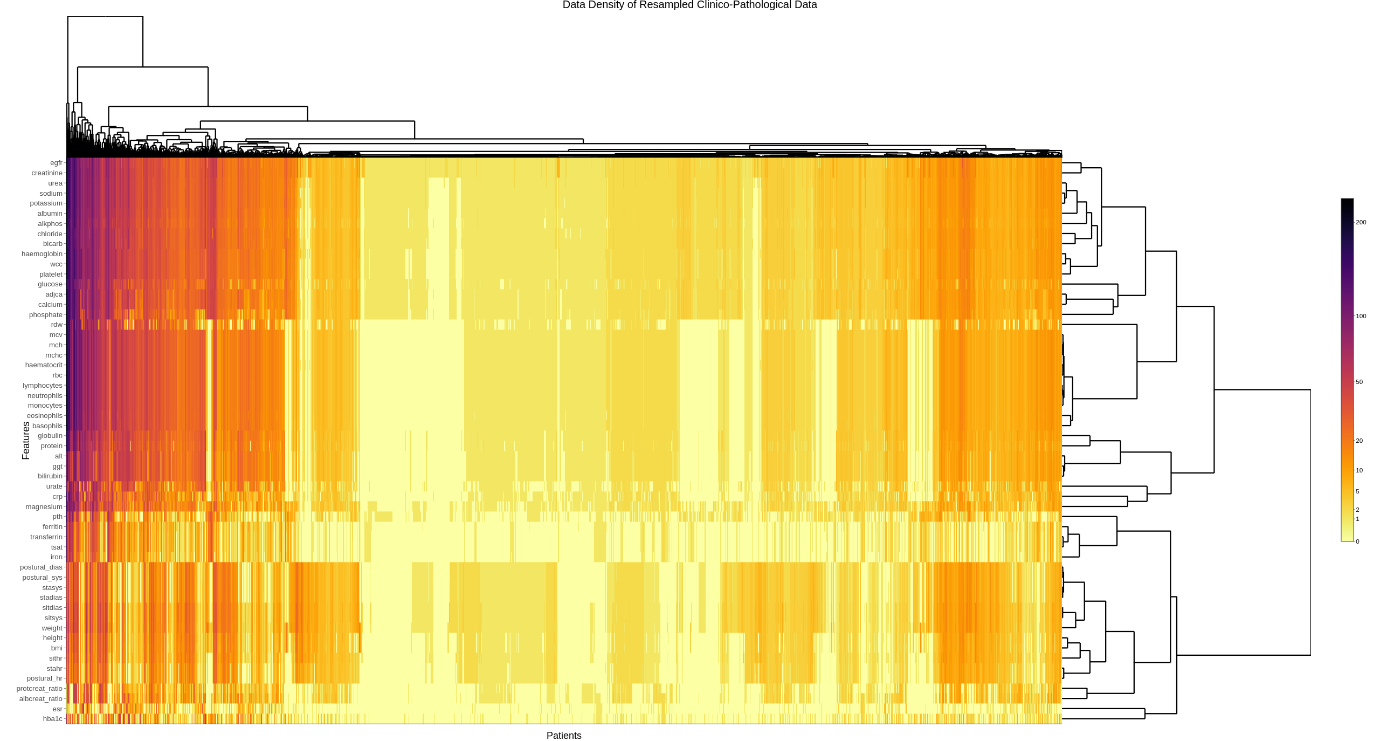


1C. Heatmap of dense3 data


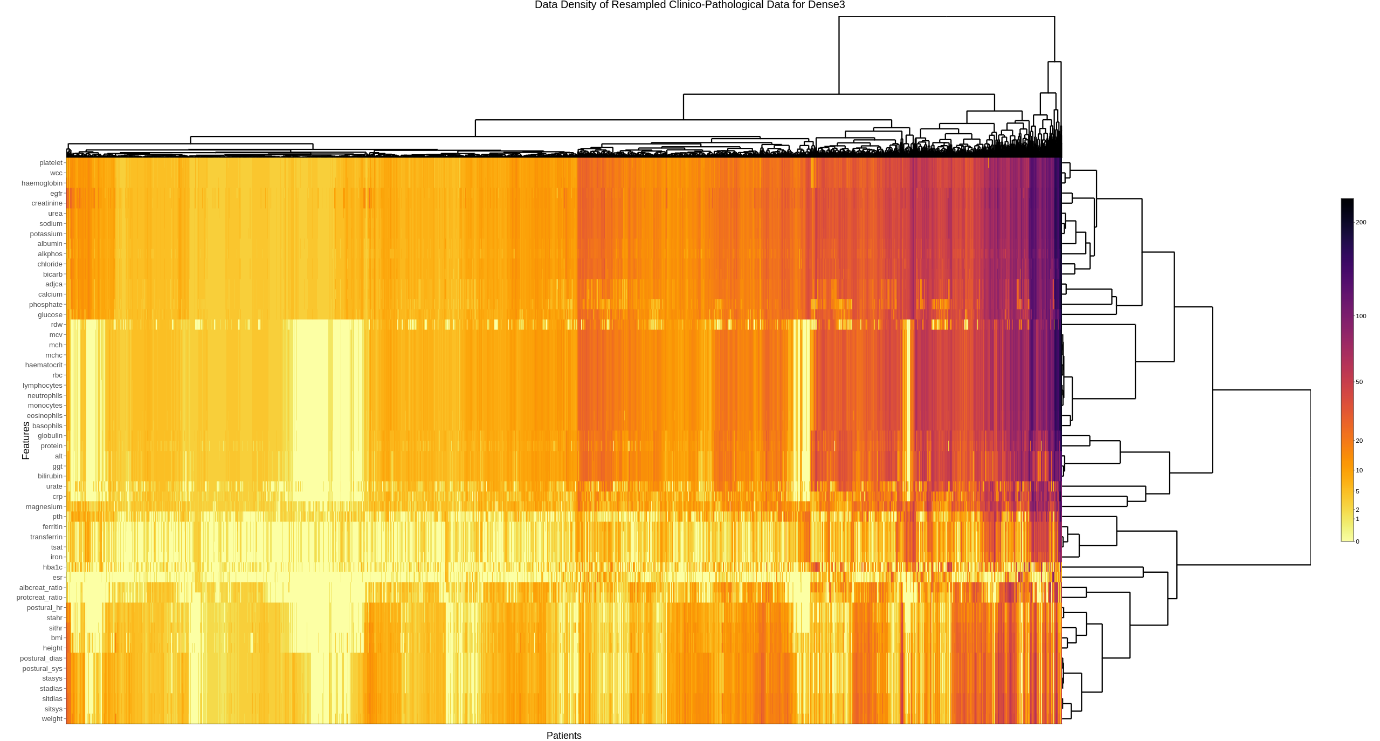


Heatmap shows the frequency (represented by the color gradient) of each test performed (y-axis) on each patient (x-axis)

Figure 2. Sensitivity analysis

2A. Concordance index

*2A.1 ESKD*


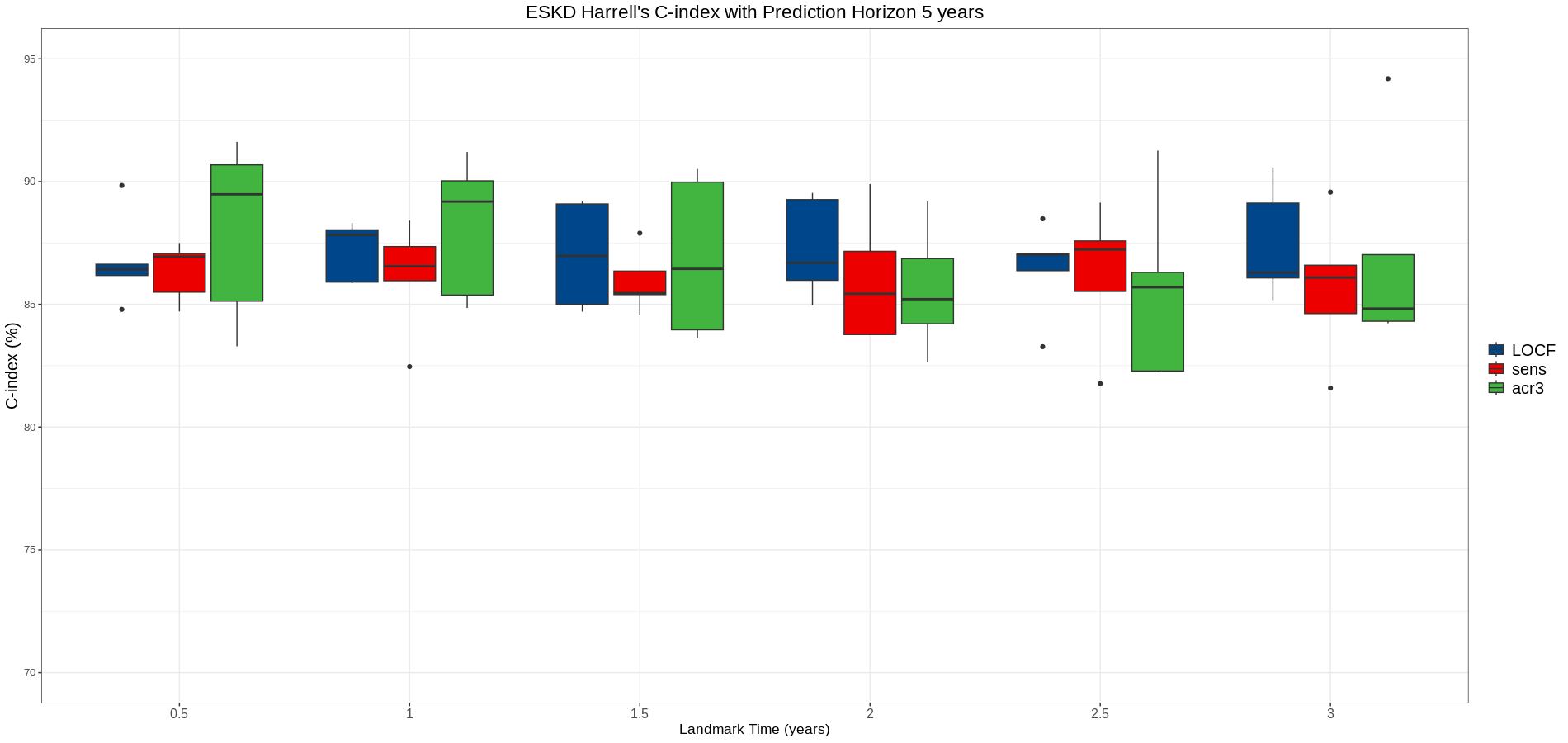


Note: Kruskal-Wallis test comparing the ESKD concordance index of the models for each landmark time was non-significant.

*2A.2 Death*


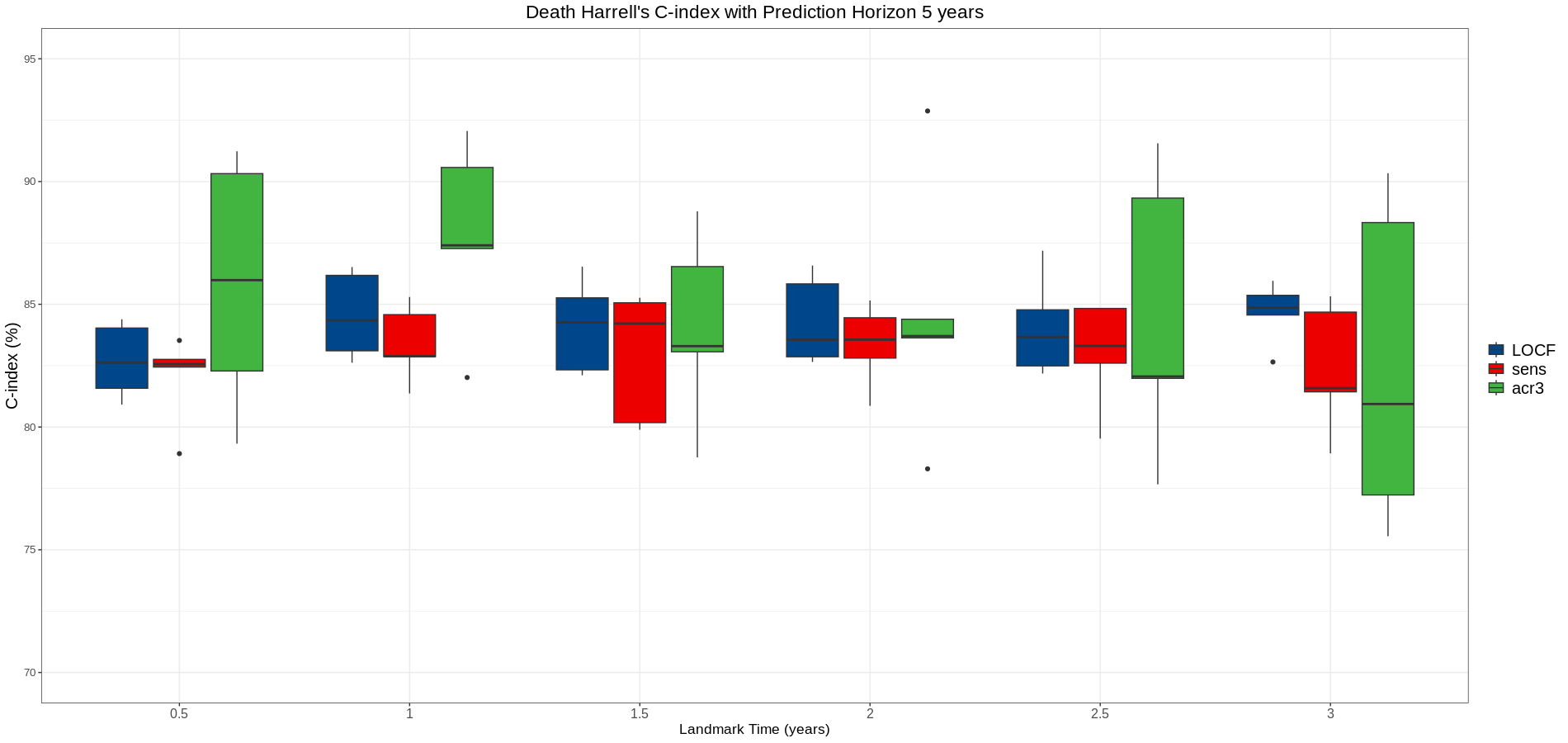


Note: Kruskal-Wallis test comparing the Death concordance index of the models for each landmark time was non-significant.

2B. Integrated Brier score

*2B.1 ESKD*


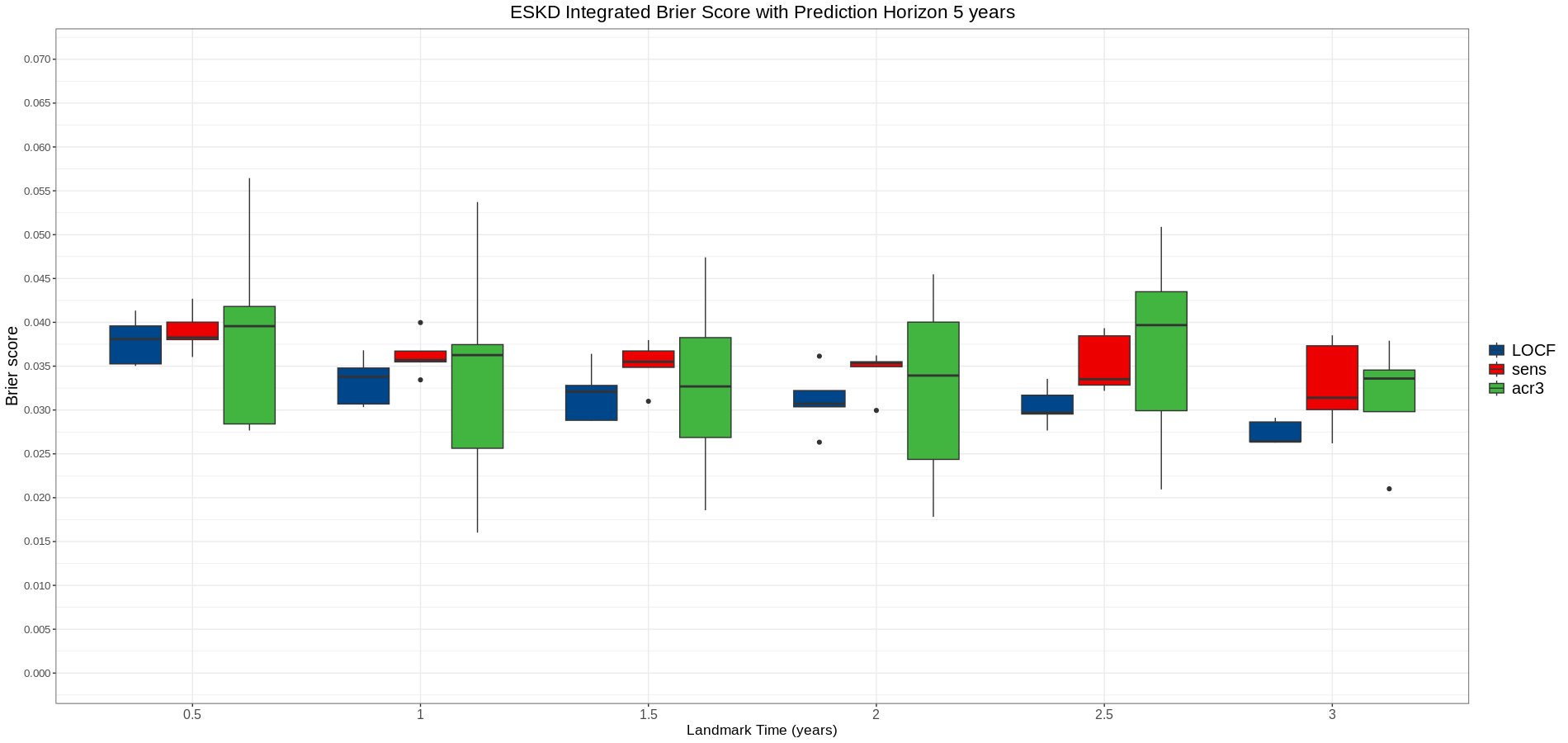


Note: Kruskal-Wallis test comparing the ESKD Integrated Brier Score of the models for each landmark time was non-significant.

*2B.2 Death*


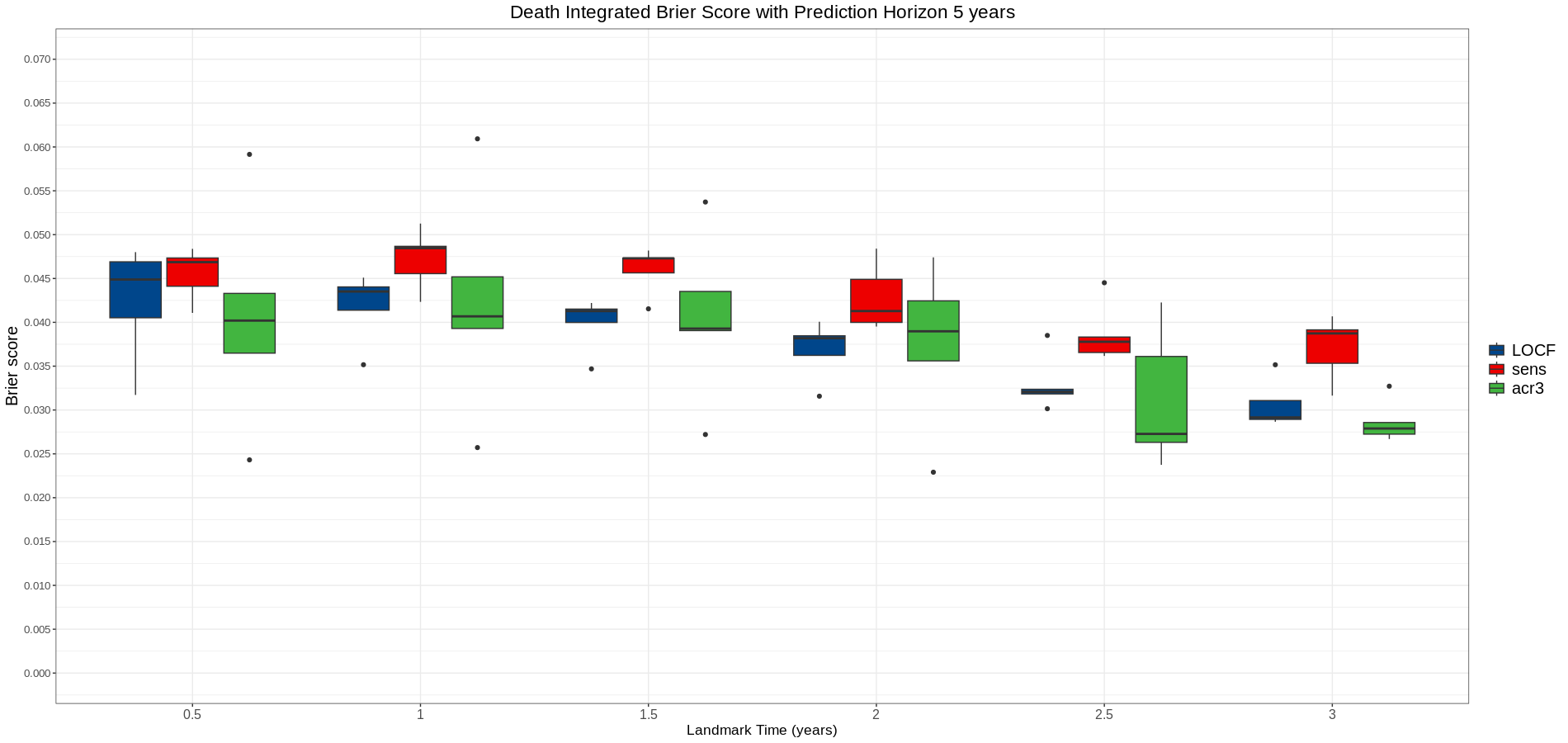


Note: Kruskal-Wallis test comparing the Death Integrated Brier Score of the models for each landmark time was non-significant.

**Supplementary Tables**

Table 1. Clinicopathological Data and Patient Demographics

| **Baseline Data** | **Unit** |
| --- | --- |
| Initial age at presentation* | years |
| Gender* | Not applicable |
| Glomerulonephritis or vasculitis diagnosis* | Not applicable |

| **Longitudinal Data** | **Unit** | **Median Frequency (IQR)** |
| --- | --- | --- |
| Adjusted serum calcium | mmol/L | 3 (1 to 12) |
| Basophil count | x10^9^/L | 2 (1 to 10) |
| Body mass index | kg/m^2^ | 2 (0 to 6) |
| Eosinophil count | x10^9^/L | 2 (1 to 10) |
| Erythrocyte sedimentation rate | mm/hour | 0 (0 to 1) |
| Haematocrit | % | 2 (1 to 10) |
| Haemoglobin* | g/L | 3 (1 to 13) |
| Haemoglobin A1c | % | 0 (0 to 2) |
| Height | meter | 2 (0 to 6) |
| Lymphocyte count | x10^9^/L | 2 (1 to 10) |
| Mean corposcular haemoglobin | fL | 2 (1 to 10) |
| Mean corposcular volume | Pg | 2 (1 to 10) |
| Mean corpuscular hemoglobin concentration | g/L | 2 (1 to 10) |
| Monocyte count | x10^9^/L | 2 (1 to 10) |
| Neutrophil count | x10^9^/L | 2 (1 to 10) |
| Platelet count* | x10^9^/L | 3 (1 to 13) |
| Postural diastolic | mmHg | 2 (1 to 6) |
| Postural heart rate | beats per minute | 2 (0 to 5) |
| Postural systolic | mmHg | 2 (1 to 6) |
| Red blood cell count | x10^12^/L | 2 (1 to 10) |
| Red cell distribution width | % | 1 (0 to 7) |
| Serum alanine transaminase | U/L | 2 (1 to 9) |
| Serum albumin* | g/L | 4 (1 to 13) |
| Serum alkaline phosphatase* | U/L | 3 (1 to 13) |
| Serum bicarbonate* | mmol/L | 4 (1 to 13) |
| Serum bilirubin | µmol/L | 2 (1 to 9) |
| Serum calcium* | mmol/L | 3 (1 to 12) |
| Serum chloride* | mmol/L | 4 (1 to 13) |
| Serum c-reactive protein | mg/L | 2 (0 to 6) |
| Serum creatinine | µmol/L | 4 (2 to 15) |
| Serum eGFR* | mL/min/1.73m^2^ | 4 (2 to 14) |
| Serum erythrocyte sedimentation rate | mm/hour | 0 (0 to 1) |
| Serum ferritin | microg/L | 0 (0 to 2) |
| Serum gamma-glutamyl transferase | U/L | 2 (1 to 9) |
| Serum globulin | g/L | 2 (1 to 10) |
| Serum glucose* | mmol/L | 3 (1 to 11) |
| Serum iron | µmol/L | 0 (0 to 2) |
| Serum magnesium | mmol/L | 2 (1 to 7) |
| Serum parathyroid hormone | pmol/L | 1 (0 to 3) |
| Serum phosphate* | mmol/L | 3 (1 to 10) |
| Serum potassium* | mmol/L | 4 (1 to 14) |
| Serum protein | g/L | 2 (1 to 9) |
| Serum sodium* | mmol/L | 4 (1 to 14) |
| Serum transferrin | µmol/L | 0 (0 to 2) |
| Serum urate | µmol/L | 1 (0 to 6) |
| Serum urea | mmol/L | 4 (1 to 14) |
| Sitting diastolic | mmHg | 2 (1 to 7) |
| Sitting heart rate | beats per minute | 2 (0 to 5) |
| Sitting systolic | mmHg | 2 (1 to 7) |
| Standing diastolic | mmHg | 2 (1 to 6) |
| Standing heart rate | beats per minute | 2 (0 to 5) |
| Standing systolic | mmHg | 2 (1 to 6) |
| Transferrin saturation | % | 0 (0 to 2) |
| Urine albumin creatinine ratio** | mg/mmol | 1 (0 to 4) |
| Urine protein creatinine ratio | mg/mmol | 1 (0 to 3) |
| Weight | Kilogram | 2 (1 to 7) |
| White cell count* | x10^9^/L | 3 (1 to 13) |

*included for model training (dense3) **included for sensitivity analysis (acr3, see Methods and Results)

Table 2. Baseline missing data

| **Variables** | **dense3**  **(N = 4,950)** | **acr3**  **(N = 2,916)** | **External cohort**  **(N = 8729)** |
| --- | --- | --- | --- |
| Serum albumin | 698 (14%) | 355 (12%) | 1370 (16%) |
| Serum alkaline phosphatase | 765 (15%) | 402 (14%) | 1497 (17%) |
| Serum bicarbonate | 947 (20%) | 558 (19%) | 4 (0%) |
| Serum calcium | 1,165 (24%) | 713 (25%) | 5956 (68%) |
| Serum chloride | 922 (19%) | 538 (19%) | 55 (1%) |
| eGFR | 0 | 0 | 0 |
| Serum glucose | 1,352 (27%) | 918 (32%) | 7583 (87%) |
| Haemoglobin | 838 (17%) | 470 (16%) | 5841 (67%) |
| Serum phosphate | 1,514 (31%) | 1,014 (35%) | 6547 (75%) |
| Platelet count | 862 (18%) | 482 (17%) | 5867 (67%) |
| Serum potassium | 527 (11%) | 235 (8%) | 5 (0%) |
| Serum sodium | 517 (11%) | 229 (8%) | 2 (0%) |
| White cell count | 853 (17%) | 475 (16%) | 5865 (67%) |
| Urine albumin creatinine ratio | NA | 2,101 (72%) | 7907 (91%) |

Abbreviation: NA, not applicable

Table 3. Performance comparison

3A. Concordance index

| **LM** | **Event** | **dense3 LOCF** | **dense 3 LME** | **dense3 LME Poly** | **dense3 LOCF Top 10** | **dense3 LOCF Top 5** | **External** |
| --- | --- | --- | --- | --- | --- | --- | --- |
| 0.5 | ESKD | 86.42 (86.18 – 86.63) | 83.35 (80.14 – 85.63) | 85.65 (84.29 – 86.18) | 86.23 (85.72 – 87.41) | 84.89 (83.97 – 86.41) | 88.3 |
| 0.5 | Death | 82.63 (81.58 – 84.03) | 81.86 (81.45 – 83.83) | 82.22 (81.7 – 84.32) | 83.71 (83.65 – 83.86) | 82.68 (82.04 – 82.72) | NA |
| 1 | ESKD | 87.82 (85.91 – 88.03) | 83.19 (81.57 – 84.54) | 84.9 (83.58 – 84.98) | 86.13 (85.69 – 86.42) | 84.78 (84.72 – 85.45) | 86.7 |
| 1 | Death | 84.34 (83.11 – 86.18) | 83.94 (83.65 – 84.06) | 84.01 (83 – 84.87) | 85.3 (85.16 – 87.73) | 84.39 (83.48 – 86.26) | NA |
| 1.5 | ESKD | 86.97 (85.01 – 89.09) | 84.15 (82.92 – 85.19) | 84.6 (81.61 – 86.42) | 84.60 (84.1 – 86.52) | 83.81 (83.3 – 87.02) | 87.1 |
| 1.5 | Death | 84.26 (82.33 – 85.26) | 83.57 (81.64 – 84.27) | 83.77 (82.64 – 84.65) | 85.07 (84.07 – 86.93) | 84.23 (83.21 – 85.97) | NA |
| 2 | ESKD | 86.69 (85.98 – 89.27) | 83.37 (81.47 – 84.48) | 86.16 (83.5 – 86.8) | 84.87 (84.21 – 87.17) | 84.66 (83.45 – 86.39) | 85.2 |
| 2 | Death | 83.55 (82.86 – 85.83) | 83.83 (81.93 – 85) | 83.98 (83.97 – 85.26) | 84.25 (84.12 – 85.78) | 83.85 (82.59 – 84.94) | NA |
| 2.5 | ESKD | 87.03 (86.37 – 87.05) | 82.52 (81.78 – 83.77) | 83.91 (83.9 – 85.49) | 84.72 (83.62 – 85.05) | 84.89 (84.46 – 85.24) | 83.1 |
| 2.5 | Death | 83.67 (82.49 – 84.78) | 82.33 (82 – 84.23) | 83.29 (83.2 – 84.15) | 83.62 (82.76 – 86.24) | 84.97 (84.59 – 85.9) | NA |
| 3 | ESKD | 86.29 (86.07 – 89.12) | 82.74 (81.53 – 83.93) | 83.96 (83.89 – 87.07) | 85.30 (83.24 – 85.85) | 85.03 (84.71 – 85.36) | 86.6 |
| 3 | Death | 84.86 (84.56 – 85.37) | 84.73 (83.09 – 85.04) | 84.7 (83.77 – 85.75) | 84.89 (84.36 – 86.44) | 84 (83.5 – 84.5) | NA |

*LM : landmark times ** values are median (IQR)

Abbreviation: NA, not applicable (see Methods)

3B. Integrated Brier score

| **LM** | **Event** | **dense3 LOCF** | **dense 3 LME** | **dense3 LME Poly** | **dense3 LOCF Top 10** | **dense3 LOCF Top 5** |
| --- | --- | --- | --- | --- | --- | --- |
| 0.5 | ESKD | 0.038 (0.035 -0.04) | 0.044 (0.042 – 0.046) | 0.041 (0.04 – 0.041) | 0.036 (0.034 – 0.038) | 0.037 (0.035 – 0.038) |
| 0.5 | Death | 0.045 (0.041 – 0.047) | 0.044 (0.04 – 0.046) | 0.044 (0.039 – 0.046) | 0.039 (0.036 – 0.039) | 0.039 (0.037 – 0.04) |
| 1 | ESKD | 0.034 (0.031 – 0.035) | 0.039 (0.035 – 0.04) | 0.037 (0.034 – 0.038) | 0.033 (0.032 – 0.034) | 0.033 (0.032 – 0.034) |
| 1 | Death | 0.044 (0.041 – 0.044) | 0.043 (0.042 – 0.044) | 0.043 (0.043 – 0.044) | 0.038 (0.035 – 0.039) | 0.039 (0.036 – 0.039) |
| 1.5 | ESKD | 0.032 (0.029 – 0.033) | 0.037 (0.035 – 0.038) | 0.037 (0.032 – 0.038) | 0.031 (0.03 – 0.031) | 0.03 (0.029 – 0.031) |
| 1.5 | Death | 0.041 (0.04 – 0.042) | 0.041 (0.041 – 0.042) | 0.042 (0.041 – 0.042) | 0.036 (0.036 – 0.037) | 0.036 (0.036 – 0.037) |
| 2 | ESKD | 0.031 (0.03 – 0.032) | 0.035 (0.035 – 0.036) | 0.034 (0.033 – 0.034) | 0.031 (0.03 – 0.031) | 0.031 (0.03 – 0.031) |
| 2 | Death | 0.038 (0.036 – 0.038) | 0.038 (0.036 – 0.039) | 0.038 (0.036 – 0.038) | 0.034 (0.032 – 0.034) | 0.035 (0.034 – 0.035) |
| 2.5 | ESKD | 0.03 (0.03 – 0.032) | 0.034 (0.034 – 0.038) | 0.032 (0.032 – 0.035) | 0.03 (0.029- 0.031) | 0.029 (0.029 – 0.03) |
| 2.5 | Death | 0.032 (0.032 – 0.032) | 0.032 (0.032 – 0.032) | 0.032 (0.032 – 0.032) | 0.029 (0.028 – 0.03) | 0.029 (0.028 – 0.029) |
| 3 | ESKD | 0.026 (0.026 – 0.029) | 0.031 (0.031 – 0.033) | 0.029 (0.029 – 0.031) | 0.027 (0.026 – 0.028) | 0.027 (0.026 – 0.028) |
| 3 | Death | 0.029 (0.029 – 0.031) | 0.029 (0.029 – 0.029) | 0.029 (0.029 – 0.03) | 0.026 (0.026 – 0.028) | 0.027 (0.026 – 0.027) |

*LM : landmark times ** values are median (IQR)

3C. Area Under the Curve for Time-dependent ROC

| **LM** | **Event** | **dense3 LOCF Top 5** |
| --- | --- | --- |
| 0.5 | ESKD | 81.8 (81.7 – 86.5) |
| 0.5 | Death | 82.4 (81.8 - 84 |
| 1 | ESKD | 84.6 (83.9 – 85.3) |
| 1 | Death | 84.9 (82.4 – 86.8) |
| 1.5 | ESKD | 84.9 (82.5 – 85.5) |
| 1.5 | Death | 84.9 (81.5 – 85.6) |
| 2 | ESKD | 85.8 (85.2 – 87) |
| 2 | Death | 84.8 (83 – 86.3) |
| 2.5 | ESKD | 86.1 (85.3 – 88.2) |
| 2.5 | Death | 85.8 (84.4 – 86) |
| 3 | ESKD | 87.5 (87.3 – 90) |
| 3 | Death | 84.7 (84.2 – 85.5) |

*LM : landmark times ** values are median (IQR)

Table 4. Median VIMP for combined ESKD and Death for all landmark times

| **Predictors** | **Median VIMP** |
| --- | --- |
| eGFR | 29.072071 |
| Initial age at presentation* | 15.814672 |
| Serum chloride | 6.020488 |
| Serum albumin | 4.74487 |
| Serum bicarbonate | 3.427642 |
| Haemoglobin | 3.133013 |
| Serum alkaline phosphatase | 2.61439 |
| Serum sodium | 2.539591 |
| Platelet count | 2.244881 |
| Serum potassium | 2.208003 |
| White cell count | 2.182732 |
| Serum calcium | 1.657719 |
| Serum glucose | 1.45479 |
| Serum phosphate | 1.395363 |
| Glomerulonephritis or vasculitis diagnosis* | 1.155806 |
| Gender* | 0.434903 |

*Baseline, non-longitudinal predictors

Table 5. Sensitivity analysis

5A. Baseline summary

| **Variables** | **dense3**  **(N = 4,950)** | **sens3**  **(N = 4,225)** | **acr3**  **(N = 2,916)** |
| --- | --- | --- | --- |
| Demographic data | | | |
| Gender - Female | 2,217 (45%) | 1,920 (45%) | 1,280 (44%) |
| Initial age of presentation (median, IQR) | 62 (47 to 73) | 65 (50 to 74) | 59 (45 to 70) |
| Glomerulonephritis or vasculitis diagnosis | 500 (10%) | 422 (10%) | 445 (15%) |
| Follow-up time in years (median, IQR) | 7 (3, 13) | 5.16 (2.34 to 8.72) | 8 (4 to 14) |
| Event | | | |
| ESKD | 1,270 (25%) | 621 (15%) | 639 (22%) |
| Death | 733 (15%) | 711 (17%) | 278 (9%) |
| Censored | 2,947 (60%) | 2,893 (68%) | 1,999 (69%) |
| Clinicopathological data | | | |
| Serum albumin in g/L (median, IQR) | 42 (39 to 44) | 42 (39 to 44) | 42 (39 to 44) |
| Serum alkaline phosphatase in U/L (median, IQR) | 78 (63 to 97) | 77 (62 to 96) | 77 (63 to 95) |
| Serum bicarbonate in mmol/L (median, IQR) | 23 (21 to 26) | 23 (21 to 25) | 24 (22 to 26) |
| Serum calcium in mmol/L (median, IQR) | 2.36 (2.27 to 2.44) | 2.37 (2.29 to 2.44) | 2.36 (2.27 to 2.43) |
| Serum chloride in mmol/L (median, IQR) | 105 (102 to 107) | 105 (103 to 107) | 105 (102 to 107) |
| eGFR in mL/min/1.73m^2^ (median, IQR) | 50 (30 to 73) | 51 (35 to 75) | 57 (36 to 77) |
| Serum glucose in mmol/L (median, IQR) | 5.7 (5 to 7.2) | 5.7 (5 to 7.2) | 5.62 (5 to 7.27) |
| Haemoglobin in g/L (median, IQR) | 133 (118 to 146) | 134 (121 to 146) | 136 (122 to 148) |
| Serum phosphate in mmol/L (median, IQR) | 1.13 (0.99 to 1.28) | 1.1 (0.98 to 1.24) | 1.12 (0.99 to 1.26) |
| Platelet count in x10^9^/L (median, IQR) | 240 (198 to 290) | 236 (196 to 286) | 245 (203 to 294) |
| Serum potassium in mmol/L (median, IQR) | 4.2 (3.9 to 4.6) | 4.2 (3.9 to 4.6) | 4.3 (4 to 4.6) |
| Serum sodium in mmol/L (median, IQR) | 139 (137 to 141) | 139 (138 to 141) | 140 (138 to 141) |
| White cell count in x10^9^/L (median, IQR) | 7.3 (6 to 8.9) | 7.2 (6 to 8.8) | 7.3 (6 to 8.8) |
| Urine albumin creatinine ratio in mg/mmol (median, IQR) | NA | NA | 13 (2 to 92) |

Abbreviation: NA, not applicable

5A. Concordance index

| **LM** | **Event** | **dense3 LOCF** | **sens LOCF** | **acr3 LOCF** |
| --- | --- | --- | --- | --- |
| 0.5 | ESKD | 86.42 (86.18 – 86.63) | 86.94 (85.5 – 87.07) | 89.48 (85.13 – 90.68) |
| 0.5 | Death | 82.63 (81.58 – 84.03) | 82.56 (82.44 – 82.76) | 85.98 (82.28 – 90.32) |
| 1 | ESKD | 87.82 (85.91 – 88.03) | 86.55 (85.96 – 87.35) | 89.19 (85.38 – 90) |
| 1 | Death | 84.34 (83.11 – 86.18) | 82.88 (82.87 – 84.58) | 87.4 (87.27 – 90.57) |
| 1.5 | ESKD | 86.97 (85.01 – 89.09) | 85.46 (85.40 – 86.35) | 86.44 (83.96 – 89.98) |
| 1.5 | Death | 84.26 (82.33 – 85.26) | 84.22 (80.18 – 85.06) | 83.3 (83.07 – 86.54) |
| 2 | ESKD | 86.69 (85.98 – 89.27) | 85.43 (83.77 – 87.16) | 85.21 (84.21 – 86.86) |
| 2 | Death | 83.55 (82.86 – 85.83) | 83.56 (82.81 – 84.45) | 83.71 (83.63 – 84.39) |
| 2.5 | ESKD | 87.03 (86.37 – 87.05) | 87.23 (85.53 – 87.58) | 85.69 (82.28 – 86.3) |
| 2.5 | Death | 83.67 (82.49 – 84.78) | 83.3 (82.6 – 84.83) | 82.06 (81.98 – 89.33) |
| 3 | ESKD | 86.29 (86.07 – 89.12) | 86.09 (84.62 – 86.59) | 84.83 (84.31 – 87.02) |
| 3 | Death | 84.86 (84.56 – 85.37) | 81.58 (81.43 – 84.68) | 80.94 (77.23 – 88.33) |

*LM: landmark times ** values are median (IQR)

5b. Integrated Brier score

| **LM** | **Event** | **dense3 LOCF** | **sens LOCF** | **acr3 LOCF** |
| --- | --- | --- | --- | --- |
| 0.5 | ESKD | 0.038 (0.035 -0.04) | 0.038 (0.038 – 0.04) | 0.04 (0.028 – 0.042) |
| 0.5 | Death | 0.045 (0.041 – 0.047) | 0.047 (0.044 – 0.047) | 0.04 (0.036 – 0.043) |
| 1 | ESKD | 0.034 (0.031 – 0.035) | 0.036 (0.035 – 0.037) | 0.036 (0.026 – 0.037) |
| 1 | Death | 0.044 (0.041 – 0.044) | 0.048 (0.046 – 0.049) | 0.041 (0.039 – 0.045) |
| 1.5 | ESKD | 0.032 (0.029 – 0.033) | 0.036 (0.035 – 0.037) | 0.033 (0.027 – 0.038) |
| 1.5 | Death | 0.041 (0.04 – 0.042) | 0.047 (0.046 – 0.047) | 0.039 (0.039 – 0.044) |
| 2 | ESKD | 0.031 (0.03 – 0.032) | 0.035 (0.035 – 0.036) | 0.034 (0.024 – 0.04) |
| 2 | Death | 0.038 (0.036 – 0.038) | 0.041 (0.04 – 0.045) | 0.039 (0.036 – 0.042) |
| 2.5 | ESKD | 0.03 (0.03 – 0.032) | 0.034 (0.033 – 0.038) | 0.04 (0.03 – 0.043) |
| 2.5 | Death | 0.032 (0.032 – 0.032) | 0.038 (0.037 – 0.038) | 0.027 (0.026 – 0.036) |
| 3 | ESKD | 0.026 (0.026 – 0.029) | 0.031 (0.03 – 0.037) | 0.034 (0.03 – 0.035) |
| 3 | Death | 0.029 (0.029 – 0.031) | 0.039 (0.035 – 0.039) | 0.028 (0.027 – 0.029) |

*LM: landmark times ** values are median (IQR)

Table 6. Comparison to Kidney Failure Risk Equation

| **Metrics** | **Top 5 2 years** | **KFRE 2 years** | **P-value** | **Top 5 5 years** | **KFRE 5 years** | **P-value** |
| --- | --- | --- | --- | --- | --- | --- |
| Concordance index | 87% (83.7 – 89.6) | 91.4% (88.9-93.9) | 0.009 | 89.1% (85.5-92.7) | 91.4% (88.9-93.9) | 0.189 |

*KFRE: Kidney Failure Risk Equation (8-Variable) **values are mean (95% confidence interval)
